# Time Series Analysis and Forecasting with Automated Machine Learning on a National ICD-10 Database

**DOI:** 10.3390/ijerph17144979

**Published:** 2020-07-10

**Authors:** Victor Olsavszky, Mihnea Dosius, Cristian Vladescu, Johannes Benecke

**Affiliations:** 1 Department of Dermatology, Venereology and Allergy, University Medical Center and Medical Faculty Mannheim, University of Heidelberg, and Center of Excellence in Dermatology, Theodor-Kutzer-Ufer 1–3, 68167 Mannheim, Germany; Victor.Olsavszky@medma.uni-heidelberg.de (V.O.); johannes.benecke@umm.de (J.B.); 2 National School of Public Health Management and Professional Development, Str. Vaselor, nr. 31, 030167 Bucharest, Romania; MDosius@snspms.ro; 3 University of Medicine and Pharmacy Victor Babes, Piaţa Eftimie Murgu, nr.2, 300041 Timisoara, Romania

**Keywords:** automated machine learning, deep learning, artificial intelligence, deadliest diseases, time series, disease prediction

## Abstract

The application of machine learning (ML) for use in generating insights and making predictions on new records continues to expand within the medical community. Despite this progress to date, the application of time series analysis has remained underexplored due to complexity of the underlying techniques. In this study, we have deployed a novel ML, called automated time series (AutoTS) machine learning, to automate data processing and the application of a multitude of models to assess which best forecasts future values. This rapid experimentation allows for and enables the selection of the most accurate model in order to perform time series predictions. By using the nation-wide ICD-10 (International Classification of Diseases, Tenth Revision) dataset of hospitalized patients of Romania, we have generated time series datasets over the period of 2008–2018 and performed highly accurate AutoTS predictions for the ten deadliest diseases. Forecast results for the years 2019 and 2020 were generated on a NUTS 2 (Nomenclature of Territorial Units for Statistics) regional level. This is the first study to our knowledge to perform time series forecasting of multiple diseases at a regional level using automated time series machine learning on a national ICD-10 dataset. The deployment of AutoTS technology can help decision makers in implementing targeted national health policies more efficiently.

## 1. Introduction

Accurate disease forecasts can help medical organizations in taking countermeasures and advance preparedness of hospitals and the general population. Recently, machine learning (ML) techniques are being increasingly implemented in the analysis of healthcare data [1]. ML analysis can help combat diseases and improve medical systems by increasing their efficiency. Particularly, deep learning, a subset of ML, has been extensively deployed over the past years due to increasing computer processing power and the availability of so-called big data sets [2,3]. Deep learning (DL) algorithms are able to perform highly complex computational analysis of massive labeled and unlabeled raw data [4]. While such DL applications have already been widely used as diagnostic tools either in disease predictions, [5,6,7] or in clinical [8,9] or pathological image analysis [10,11], there is limited ML deployment described for time series forecasting in the current literature [12].

Since epidemics or pandemics are known to cause not only individual, but also societal damages [13,14,15], the majority of disease forecast models have been created for infectious diseases [12,16,17,18]. Some of the most forecasted diseases have been influenza [16,19,20,21,22,23], hand, foot, and mouth disease [17,24,25,26], and tuberculosis [18,27,28]. The need for infectious disease predictions was mainly attributed to delayed responses of medical organizations to combat such health threats [12]. In order to predict infectious disease trends in real time, some authors included nonmedical databases such as common internet search queries [23], Google Trends [19,29,30], or Twitter [22,31]. Nevertheless, web-based disease tracking methods are considered mere appositions rather than substitutes for classical epidemiologic databases [32,33]. Even if conventional medical datasets have delayed reports, the advancement in ML techniques have helped build increasingly accurate forecast models over the last ten years [16]. While some of the first forecasting analyses employed generalized linear model forms of regression analysis, such as Poisson regression models [34], the SARIMA (seasonal autoregressive integrated moving average) model developed from the autoregressive integrated moving average (ARIMA) model became one of the most used forecasting models [26,27]. Beyond SARIMA, other widely used models include the generalized linear regression (GLM) model and Bayesian networks [20]. By combining linear regression models [25] and comparing them to deep neural network (DNN) and long-short term memory (LSTM) learning models, it soon became obvious that DL models were superior to linear models such as SARIMA [12,23]. Furthermore, employment of hybrid models, such as ARIMA-NARNN (nonlinear autoregressive neural network), did not improve the modeling performance when compared to single SARIMA or NARNN model applications [35]. In summary, most published time series forecasting analyses had various limitations. Either deep learning algorithms were not used [19,21,29,30,33,36], the datasets were small [12,20,22,23,31], or the used computational software could not simultaneously employ a higher number of models [12,37]. Moreover, the global demand for machine learning solutions often exceeds the expertise of healthcare providers to effectively utilize ML [38].

Despite the focus on deep learning algorithms or classically inspired approaches such as SARIMA or NARNN, other powerful ML algorithms can be successfully used in time series forecasting; however, they are not naturally time-aware and require substantial data preparation and feature engineering [39]. Leveraging these modern algorithms requires defining a useful historical period of time to examine candidate features, and then creating informative features based on past and present examples of the target and covariate. These can include various lags inside a range, rolling means, minimums, maximums, Bollinger Bands and statistics, and rolling entropy or rolling majority for categorical features. In addition to creating new features, transforms often need to be made on the target based on tests on the target’s stationarity, periodicity, or trend (e.g., exponential), to improve predictive performance. This may include a log-transformation for exponential trends and multiplicative models, periodic differencing to make an integrated model with stationary targets, or a simple naive difference. Data preparation of this sort allows for modern ML techniques such as XGBoost (eXtreme Gradient Boosting) and LightGBM (Light Gradient Boosting Machine), to be used, which again presents another challenge and layer of decisions to be made for researchers wishing to use these techniques, as the models’ hyperparameters must be optimized, the model code implemented, and the models trained and validated appropriately on historical data. Automated time series machine learning (AutoTS) addresses these challenges by empirically testing and evaluating dozens or hundreds of combinations of data preprocessing steps and algorithms to select the model with the least model error on out-of-sample data.

This study aimed to perform predictive time series analysis using a national ICD-10 dataset of Romania over the period 2008–2018 with AutoTS. Using an AutoTS platform [39,40], we have predicted the incidence of the ten deadliest diseases in Romania, as defined by the WHO (World Health Organization) [41], consisting of ischemic heart diseases, stroke, chronic obstructive pulmonary disease, lower respiratory infections, Alzheimer’s disease, lung cancer, diabetes mellitus, road injuries, diarrheal diseases, and tuberculosis. For each affliction, we have selected the most accurate ML model and predicted the monthly counts of new cases for every NUTS 2 region of Romania [42].

To our knowledge, there has been no AutoTS forecasting analysis of an ICD-10 medical dataset, on a national or even multinational scale. The goal of this project is to perform highly accurate predictive analyses, in order to improve disease prevention, reduce medical costs, and allow officials to allocate resources effectively in response to public health issues.

## 2. Materials and Methods

### 2.1. Data Selection

Romania started using the US DRG (diagnosis-related group) mechanism for hospital reporting in 2003 [43]. Switching towards an Australian system, the current version adopted by Romania uses the same International Classification of the Diseases, which made the data compatible for analysis and comparison for the entire period of time [44]. Data from the National DRG Database is reported monthly to the National School of Public Health, Management and Professional Development (NSPHMPDB) in Bucharest. Over a period of 11 years, starting from 2008 until 2018, all hospitalized patients in Romania classified into a diagnosis-related group (DRG) [45] were included in the database [46].

### 2.2. Data Preparation and Extraction

Data was prepared from the primary national database using Paxata in the DataRobot platform [47]. Datasets per analyzed affliction were extracted on a regional NUTS 2 level according to the corresponding ICD-10 codes provided by NSPHMPDB (Table S1). Disease codes were searched and validated with the WHO ICD-10 online application [48] and the “ICD-10_AM diagnosis and procedures list” provided by the National School of Public Health, Management and Professional Development (NSPHMPDB). Only new hospitalized cases with targeted diseases recorded as the main diagnosis were selected and aggregated into new cases per month per NUTS 2 region. The necessary features for creating a secondary time series database are shown in the flow chart of the study selection process (Figure S1). Data is not normalized during data preparation. We further enriched the dataset with the number of working days in the month and the total number of days in the month, as well as a calendar of events of 26 public holidays (Table S2). The secondary time series database is deposited online (https://www.synapse.org/#!Synapse:syn22242698).

### 2.3. Experimental Setup

After uploading each time series dataset onto the AutoTS platform [49] and selecting the appropriate forecasting target, i.e., “new cases”, a time frame needed to be set to define a rolling window to derive descriptive features relative to the Forecast Point, i.e., the time the prediction is being made. This so-called Derivation Window was empirically tested using 4, 6, 8, 10, and 12 months before the forecast point for each disease. The Derivation Window that produced models with the lowest Gamma Deviance was chosen (Table 1). A Forecast Window (FW) defines the range of future values chosen to be predicted relative to the Forecast Point, called Forecast Distances (FDs). A FD of 24 months was used for each disease. After defining the modeling project settings and target, a model fitting procedure of preprocessing, algorithms, and postprocessing steps was performed by the AutoTS tool (Figure S2). The AutoTS platform simplifies model development by performing a parallel heuristic search for the best model or ensemble of models, based on both the characteristics of the data and the prediction target. During the modeling process, many independent challenger models are developed. The time series functionality works by encoding time-sensitive components (such as lags and moving averages) as features, transforming the original input dataset into a modeling dataset that can use conventional machine learning techniques. The AutoTS tool automatically creates and selects time series features in the modeling data and automatically detects whether a project’s target value is stationary. If the target is not stationary, or shows strong seasonality, it attempts to make it stationary by applying a differencing strategy prior to modeling, thus improving the accuracy and robustness of the underlying models. Next, a series identifier is defined as the NUTS 2 hospital region. More precisely, a column containing the NUTS 2 region of the hospital must be identified, so that the different timepoints of a disease can be attributed to their corresponding NUTS 2 region. This tells the AutoTS tool that there are multiple subsets of data to model and evaluate in the dataset. Importantly, having multiple series allows the algorithms to learn effects that are present across the NUTS 2 regions. Finally, information about the selected target variable and predictors is used to define a set of candidate blueprints for analysis; here, blueprint stands for the combination of data preprocessing steps, transformations, and machine learning algorithm. It then trains models for each blueprint and ranks them based on a validation and holdout accuracy score (Figure S2).

### 2.4. Model Selection

In order to assess any model’s performance, out-of-time validation (OTV) is employed, which allows the selection of specific time periods to test the model stability, creating so-called data “backtests” [50]. Backtesting ensures that each algorithm is learning its parameters, or “fitting” the data, on historical examples only, and model performance is only being evaluated on unseen, “out of sample”, data in a proceeding period of time in the future. The length of training data used was 10 years for each dataset with three backtests used by the AutoTS tool (Figure S3). The validation time period for each backtest was one year for all diseases, except diabetes, road injuries, stroke, and heart disease using eight months. Across all projects, the final year of data was set aside as “holdout” data and was not evaluated until the final models were selected. The year 2018 was chosen as the holdout partition. The performances of these models are ultimately exposed, enabling the selection of the best model for the problem being addressed (Table S3).

After the data had been examined by the platform, it began the modeling process. A wide variety of models are tried by the tool, including common techniques such as SARIMA and more modern approaches such as eXtreme Gradient Boosting. On average, 29 different models were tried per disease (Table 1). The models were evaluated according to a number of metrics, including Gamma Deviance, mean absolute error (MAE), and mean absolute percentage error (MAPE). These scores were available for the first backtest, average of all backtests, and the holdout portion of the data. Ultimately, the top performing models were chosen based on the score of the optimization metric chosen by the platform, either Gamma Deviance or root mean square error (RMSE), on the holdout portion of the data. RMSE is a frequently used goodness-of-fit statistic, which summarizes the discrepancy between observed values and the values expected under the model in question. It is a good measure of how accurately the model predicts the response, and it is the most important criterion for fit if the main purpose of the model is prediction. Deviance is another goodness-of-fit statistic for models using the sum of squares of residuals (Gamma) in ordinary least squares to cases where model-fitting is achieved by maximum likelihood. After the model was identified, it was further refined by examining permutation-based feature importance. Features that contributed to the performance of the model were kept, while all other features were dropped. The model was run again using the new feature list, and if it performed better than the previous version of the model, it was selected as the final model (Table S4).

### 2.5. Forecasting

To extract predictions out of the best performing model, first the model is retrained up to the last month available in the dataset, using the same hyperparameters. Next, using the most recent observations as defined by the derivation window, predictions can be obtained from this updated model, which now consists of the estimated values of new cases per NUTS hospital region for each of the 24 months in the forecast window.

### 2.6. Ethics Statement

This study was reviewed and approved by the ethics committee of the National School of Public Health, Management and Professional Development (NSPHMPDB) from Bucharest, Romania (4854-04.11.2019) and by the Medical Ethics Committee II of the Medical Faculty Mannheim, Heidelberg University (2019-873R), Germany.

## 3. Results

### 3.1. Retrospective Analysis of the Ten Leading Causes of Death in Romania over the Period 2008–2018

In order to perform time series forecasting, a series of data points in time order had to be prepared for each one of the top 10 deadliest diseases, as defined by the WHO [41]. For this purpose the corresponding ICD-10 codes for ischemic heart diseases, stroke, chronic obstructive pulmonary disease, lower respiratory infections, Alzheimer’s disease, lung cancer, diabetes mellitus, road injuries, diarrheal diseases, and tuberculosis (Table S1) were extracted from the whole ICD-10 data set of hospitalized patients in Romania from the period 2008–2018. Since the aim of the study was to predict future new cases of each disease, only the ICD-10 codes used as main diagnoses in the data set were employed. We have deliberately not included ICD-10 codes categorized as secondary diagnoses, since physicians often tend to encode recoveries, anamnestic recalls, or unproven diagnoses in this category [51]. Hence, new cases of each disease represent the absolute count of every main diagnosis that necessitated a hospitalization episode. These disease counts were further classified into eight NUTS 2 regions to facilitate the detection of regional differences.

**Figure 1:**
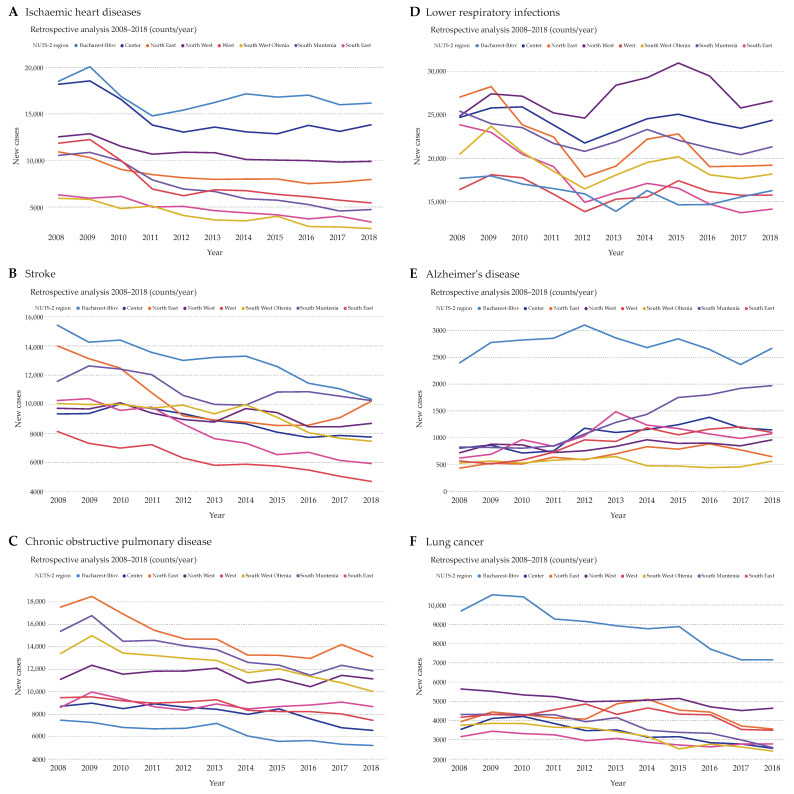
Total case counts of patients hospitalized due to ischemic heart diseases (**A**), stroke (**B**), chronic obstructive pulmonary disease (**C**), lower respiratory infections (**D**), Alzheimer’s disease (**E**), and lung cancer (**F**). Only diseases encoded as main diagnoses that facilitated hospitalization were counted for every NUTS 2 region of Romania per year over the period 2008–2018.

The retrospective analysis of ischemic heart diseases revealed an obvious decline in new cases from 2009 to 2011 in all regions (Figure 1A). A slight decrease in new cases was still observable after 2011 in most regions, apart from Bucharest-Ilfov and Center, which were also the regions with the highest numbers of ischemic heart disease hospitalizations. Stroke, on the other hand, showed a constant decline in almost all regions from 2008 to 2018, with the exception of North East, a region where the stroke counts started increasing after 2016 (Figure 1B). Overall, Bucharest-Ilfov had the highest stroke case counts. Unexpectedly, Bucharest-Ilfov revealed the lowest case counts in chronic obstructive pulmonary disease, a disease with an obvious decline in new cases in all regions (Figure 1C). Lower respiratory infections showed an alternating course of case counts in almost all regions (Figure 1D). In this case, a decline in new cases was observable from 2009 to 2012 followed by an overall, but sinusoidal, increase in new cases after 2012. Next, Alzheimer’s disease counts showed a small trend upwards, with Bucharest-Ilfov being the region with the highest and concurrently stable case counts over the years (Figure 1E). Interestingly, the second highest counts of Alzheimer’s disease were observed in South Muntenia with an obvious ascending slope. Regarding lung cancer, there was a clear decline in case counts in Bucharest-Ilfov (Figure 1F). In comparison, all other regions showed a rather small decline with at least half as many case counts compared to the Bucharest-Ilfov region.

**Figure 2:**
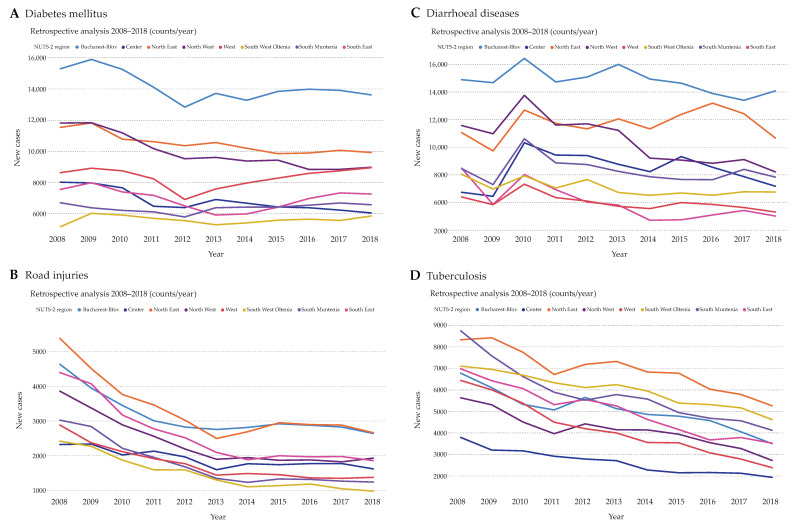
Total case counts of patients hospitalized due to diabetes mellitus (**A**), road injuries (**B**), diarrheal diseases (**C**), and tuberculosis (**D**). Only diseases encoded as main diagnoses that facilitated hospitalization were counted for every NUTS 2 region of Romania per year over the period 2008–2018.

In case of diabetes mellitus, the counts of new cases were relatively stable over the years with Bucharest-Ilfov having the highest and South West Oltenia the lowest numbers (Figure 2A). Differently, road injuries showed a clear decline from 2008 to 2018 throughout all regions with the numbers almost halving during this observation period (Figure 2B). Interestingly, Bucharest-Ilfov shared with North East the highest counts of road injuries, starting with 2015. While diarrheal diseases also showed a stable disease count with a noteworthy increase from 2009 to 2010 (Figure 2C), tuberculosis displayed a striking decline of approximately 40% over the whole period of 10 years (Figure 2D). Most tuberculosis cases were noted North East, while the Center region had the lowest counts.

### 3.2. Employment of Automated Machine Learning on the Time Series Datasets and Selection of the Most Accurate Forecasting Models

**Figure 3:**
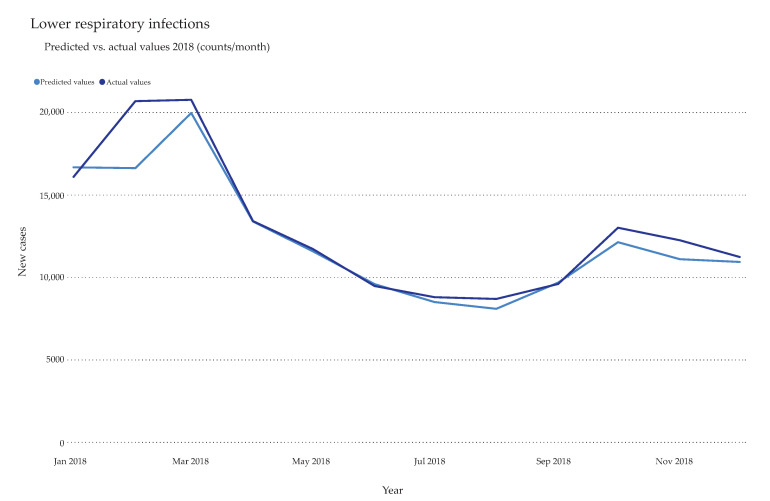
Holdout validation. Exemplary comparison between predicted total cases of lower respiratory infections and actual total cases extracted from the data set. The year 2018 was selected as holdout to test the model performance. All hospitalized lower respiratory infections cases in Romania (actual values) were plotted on a monthly basis against the predicted values calculated by the AVG Blender model (predicted values).

The year 2018 was chosen as the holdout partition (Figure 3). The holdout was not part of the training data set and only served for verifying the model. Therefore, every trained model predicted the monthly case counts for 2018 and was compared to the actual values. The top performing model for each disease was selected based on the optimization metric chosen by the platform, either Gamma Deviance or RMSE (Table S3). Other estimators, such as R-squared (coefficient of multiple determination for multiple regression), mean absolute error (MAE, average magnitude of the errors), and mean absolute percentage error (MAPE, average of the unsigned percentage error), were also taken into consideration. Notably, ensembles of multiple models, in the form of an average (AVG) blender model, yielded the lowest MAE scores in most datasets (Table 1). This model takes the predictions from several input models and averages them together into a metamodel. Predictions are made from each of the input models and ultimately combined. Other selected models included eXtreme Gradient Boosting and ElasticNet Regressor. Gradient Boosting Machines (GBMs) are a generalization of Freund and Schapire’s adaboost algorithm [52] to handle arbitrary loss functions. GBMs differ from random forests in a single major aspect: rather than fitting individual decision trees in parallel, the GBM fits each successive tree to the residual errors from all the previous trees combined. Extreme Gradient Boosting is a very efficient, parallel version of GBM that has been heavily optimized and tweaked for faster runtimes and higher predictive accuracy. ElasticNet is a linear regression model trained with L1 (Lasso regression) and L2 (ridge regression) prior as regularizer. This model is useful when there are multiple features which are correlated with one another. With the exception of tuberculosis, Alzheimer’s diseases, diarrheal disease, and road injuries, the MAPE was lower than 10% (Table 1).

### 3.3. Time Series Forecasting for the Years 2019 and 2020

**Figure 4:**
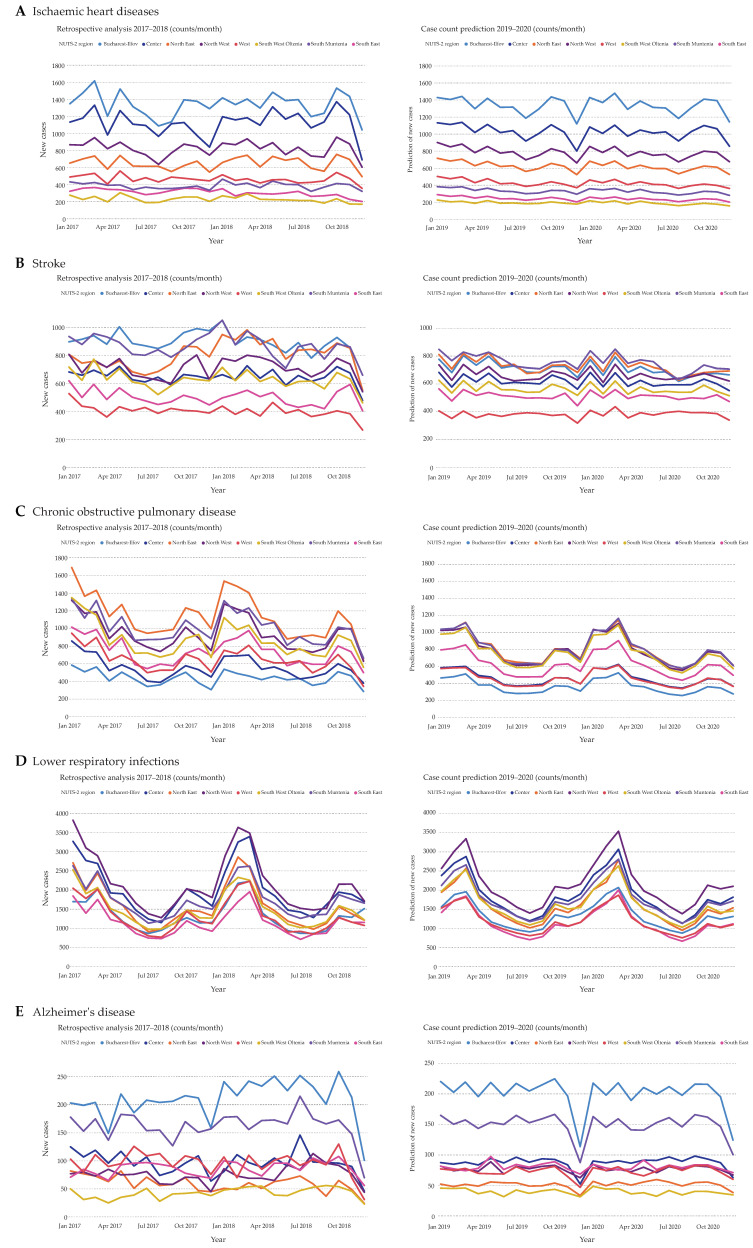
Comparison between total disease cases in the years 2017 and 2018 (left side) and predicted disease cases for the years 2019 and 2020 (right side). All hospitalized cases of ischemic heart diseases (**A**), stroke (**B**), chronic obstructive pulmonary disease (**C**), lower respiratory infections (**D**), and Alzheimer’s disease (**E**) were plotted on a monthly basis against the predicted values.

**Figure 5:**
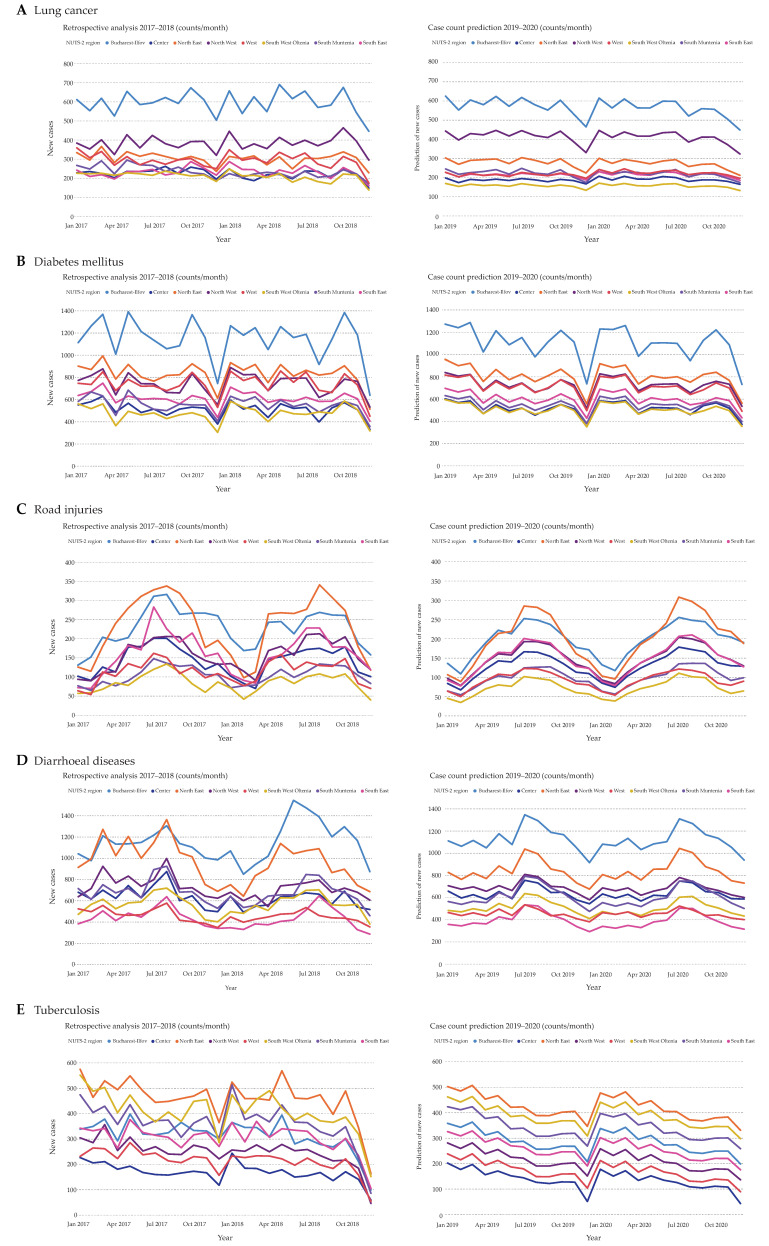
Comparison between total disease cases in the years 2017 and 2018 (left side) and predicted disease cases for the years 2019 and 2020 (right side). All hospitalized cases of lung cancer (**A**), diabetes mellitus (**B**), road injuries (**C**), diarrheal diseases (**D**), and tuberculosis (**E**) were plotted on a monthly basis against the predicted values.

For the purpose of better visualization and comprehension, disease counts of the last two years in the analyzed dataset, namely, 2017 and 2018, were plotted on a monthly basis next to the predicted counts of 2019 and 2020 (Figure 4 and Figure 5). The overall ischemic heart diseases development of new cases seems to remain stable with low fluctuations (Figure 4A). A dip in case counts was noticed during December of each year. Furthermore, there are parallel curve progressions of the predicted disease counts of every region, with Bucharest-Ilfov showing the highest case counts. While the prediction of stroke counts also shows a certain stability, South Muntenia and North East predominantly reveal the highest numbers of stroke hospitalizations (Figure 4B). Moreover, Bucharest-Ilfov showed a decline in case counts starting with 2018. Another reduction in case counts is observed with chronic obstructive pulmonary disease, especially in the North East region, when comparing the predicted years to the previous ones (Figure 4C). Furthermore, there is a peak in hospitalization episodes noticeable during wintertime in all regions for chronic obstructive pulmonary disease. Next, lower respiratory infections will retain their strong fluctuation during the years (Figure 4D). Hospitalizations due to lower respiratory infections are usually high during the first three months of each year and have the lowest counts during the summer. Noteworthy is a second relatively small peak occurring during October of each year. Regarding Alzheimer’s disease, Bucharest-Ilfov and South Muntenia have the most cases, while Center, North West, West, and South East share highly similar numbers (Figure 4E). Here, South West Oltenia remains the region with the fewest Alzheimer’s disease hospitalizations. Another dip in counts is visible in this case during December.

Lung cancer has a similar disease course to Alzheimer’s disease (Figure 5A). According to our prediction, there are no significant changes in lung cancer case counts when compared to 2017 and 2018. Moreover, the predicted case counts of diabetes mellitus are very similar to the years before (Figure 5B). Another distinct seasonal trend with the peak during summer and the highest counts in North East and Bucharest-Ilfov is seen in road injuries (Figure 5C). Here, South West Oltenia shows the lowest fluctuations of predicted counts. Interestingly, we observed a partial dependence of 37% to calendar dates. Spring holidays are associated with higher case counts of road injuries (Figure S4). Similarly, diarrheal diseases also show seasonality, with the highest counts in summer and lowest counts in winter, mainly in November and December (Figure 5D). Finally, there is yearly seasonality observed with tuberculosis, including the already known reduced hospitalization cases in December (Figure 5E). There is a stable decline when looking at tuberculosis from the beginning of 2017 onwards, which is continued throughout 2019 and 2020. The regions North East and South West Oltenia keep alternating in regard to the highest case counts.

When compared to the current literature, this is the first study on a national ICD-10 database to perform thorough time series forecasting on multiple diseases on a regional level using AutoML to select the most accurate of a multitude of models (Table S5).

## 4. Discussion

This is the first study to apply automated machine learning for time series forecasting on a nationwide ICD-10 dataset. Using data from all hospitalized patients from 2008–2018, we were able to analyze region-specific hospitalization counts for the ten deadliest diseases in Romania and perform forecasts for the years 2019 and 2020. Our findings corroborate previous studies in several important ways. Cardiovascular diseases, such as ischemic heart diseases and stroke, are the leading cause of death in Romania [53]. Western countries, for example, have managed to lower the mortality caused by ischemic heart diseases due to improvement of primary prevention and advances in diagnostic approaches [54]. Our retrospection of the last decade in Romania echoes this statement to a certain extent as well, since the hospitalizations of ischemic heart diseases and stroke showed a continuous drop from 2008 until 2018. Our predictions do not confirm this trend but show a rather stable count for these diseases for 2019 and 2020. Since there have only been analyses on Romanian macroregions [53], our NUTS 2 regional forecast could help decision makers identify specific regions with rising trends. Given that Romania has one of the highest estimated risks of developing stroke [55], and the number of new stroke cases is expected to double by 2060 [56], additional actions are mandatory in the public health sector to lower this incidence of these cardiovascular diseases.

According to the WHO, chronic obstructive pulmonary disease cases will continue to grow in the future and become the third leading cause of death by 2030 [57]. Romania has an intermediate prevalence, calculated at around 10% [58,59], and both our retrospective and forecasting analyses revealed decreasing numbers in chronic obstructive pulmonary disease hospitalizations. Lower respiratory infections, on the other hand, consistently showed seasonality, with peaks in winter and lows in summertime. This is especially important for Romania, since the influenza incidence is the highest among children aged 0–4 years [60] and the lower respiratory infections mortality rate per 100,000 people for all ages is the highest in this country when compared to the whole Balkan Peninsula [61]. Alzheimer’s disease presented a doubling in hospitalizations after 1994, with continuously growing numbers ever since [62]. While we also observed an upwards trend after 2008, our forecasting results revealed steady counts for 2019 and 2020. Importantly, Bucharest-Ilfov and South Muntenia are leading regions in Alzheimer’s hospitalizations in comparison to all other NUTS 2 regions. Similarly, high counts were seen for Bucharest-Ilfov and North West with lung cancer predictions. Despite several indications that the incidence of lung cancer would continue to rise in Romania [63,64], we observed a decline in hospitalization episodes. This might be due to nationally instituted antitobacco policies [63].

We have observed a fall in diabetes mellitus cases until 2012 and predicted constant counts. It should be noted that hospitalizations episodes do not necessarily reflect the overall incidence of a disease. This could be especially true for diabetes mellitus. While ICD-10 hospitalization case counts are relatively constant, a rise in incidence has been predicted for Romania [65]. Road injuries, on the other hand, make up almost 10% of all injuries treated in emergencies clinics or hospitalizations [66]. While Romania has managed to reduce road mortality, it still has the most road traffic fatalities in the European Union [67,68]. We even predicted the highest road-injury-related hospitalization cases in the North-East region during the summer peaks. In the case of diarrheal diseases, estimated to be the leading cause of death globally and having a declining incidence [69,70], we only noticed a slight reduction in new cases over the period 2008–2018 and predicted further stable counts. Finally, tuberculosis, with Romania known to have the highest incidence of extensively drug-resistant tuberculosis in the European Union [71], displayed promising declining hospitalization cases over the years. Starting in 2002, Romania has made significant progress in fighting the tuberculosis epidemic by implementing nationwide prevention and management programs [72]. This decreasing trend was supported with our forecast model. In line with other time-series analyses on tuberculosis that described a seasonality [18,27,28], we see a strong dip in the hospitalization curve in December, followed by a steep rise in the early months of the year. This dip is visible in most diseases, namely, ischemic heart disease, chronic obstructive pulmonary diseases, Alzheimer’s, lung cancer, diabetes mellitus, and tuberculosis, not only in the predicted months of December 2019 and 2020, but also December 2017 and 2018. This is attributed to a fall in hospitalization cases due to the winter holidays of Saint Nicholas, Christmas, and New Year’s Eve.

## 5. Conclusions

In summary, we performed an exhaustive, time-saving analysis with a nation-wide ICD-10 medical dataset encompassing a period of eleven years. Given the fact that hospitals use different applications to collect own patient data (diagnostics, blood tests etc.), which cannot be harmonized and aggregated, the ICD-10 dataset represents the only major, internationally used big dataset that can be employed for medical studies. By utilizing a novel automated machine learning tool, we could perform highly accurate predictions of the ten leading causes of death on a regional level for the whole country of Romania. While other machine learning studies usually use one model for one disease, the deployed AutoTS platform compared a multitude of models and allowed the selection of the most accurate one. It is noteworthy that the used dataset did not contain any outpatient, but only hospitalization records. Therefore, one important limitation of our study is the predicted case counts not representing the incidence of the ten analyzed causes of death, but rather the hospitalization episodes attributed to these diseases. Another selection bias could arise from inconsistencies of the primary national database, since diseases are coded by healthcare workers. Some hospitals may not have the necessary resources for training professional healthcare coders, given that some diseases have long lists of potentially relevant codes, which could lead to confusion. Nevertheless, the predicted changes in case counts and their geographic dynamics can help officials performing countermeasures, allocating resources, or raising public awareness through more aimed operations.

## Figures and Tables

**Table 1:** Selected model performance validation based on holdout.

Disease	Years	Compared Models	Selected Model	Gamma Deviance	RMSE	R-Squared	MAE	MAPE
Ischemic heart disease	DW = 6 FD = 24	25	AVG Blender	0.0110	65.6272	0.9725	48.5973	8.3663
Stroke	DW = 6 FD = 24	29	eXtreme Gradient Boosting on ElasticNet Predictions	0.0140	82.1981	0.8000	57.5654	8.8361
Chronic obstructive pulmonary disease	DW = 12 FD = 24	35	AVG Blender	0.0117	79.8697	0.9126	61.3592	8.7560
Lower respiratory infections	DW = 8 FD = 24	25	AVG Blender	0.0108	192.8462	0.9045	127.5683	7.4239
Alzheimer’s disease	DW = 8 FD = 24	28	AVG Blender	0.0579	21.7291	0.8688	16.0741	19.9726
Lung cancer	DW = 12 FD = 24	35	eXtreme Gradient Boosted Trees Regressor with Early Stopping (Gamma Loss)	0.0115	32.7293	0.9372	24.7214	8.5870
Diabetes mellitus	DW = 6 FD = 24	26	AVG Blender	0.0053	50.9413	0.9499	37.2955	5.5817
Road injuries	DW = 6 FD = 24	25	Elastic-Net Regressor (L2/Gamma Deviance) with Forecast Distance Modeling	0.0338	25.7580	0.8410	19.6105	15.1879
Diarrheal Disease	DW = 10 FD = 24	25	AVG Blender	0.0175	108.7063	0.8274	74.4832	10.7970
Tuberculosis	DW = 12 FD = 24	41	eXtreme Gradient Boosted Trees Regressor with Early Stopping	0.0674	54.8689	0.7771	36.5015	21.7094

Note. After choosing the length of training data for the backtests, Derivation Window (DW), and the length of forecasted data (FD), models were compared and validated for each disease by the AML (automated machine learning) platform. The year 2018 was chosen as holdout, and the predicted values were compared to the actual values. Model selection was based on the Gamma Deviance or root mean square error (RMSE). Other calculated estimators were R-squared, the mean absolute error (MAE) and the mean absolute percentage error (MAPE). The total number of compared models, as well as the final selected model, are listed. These final selected models were either the AVG (average) Blender, the eXtreme Gradient Boosting model or the Elastic-Net Regressor.

## Supplementary Materials

The following are available online at https://www.mdpi.com/1660-4601/17/14/4979/s1, Figure S1: Flow chart of study selection process, Figure S2: Model development workflow process (model blueprint), Figure S3: Schematic representation of model development procedure for lower respiratory infections, Figure S4: Average of hospitalizations due to road injuries on selected public holidays in Romania, Table S1: Listing of ICD-10 codes selected for data extraction and preparation from the whole ICD-10 data set of hospitalized patients in Romania during the period 2008–2018, Table S2: Romanian Public Holidays, Table S3: Exemplary listing of model performances calculated by the AML tool for lower respiratory infections, Table S4: Exemplary summary statistics for lower respiratory infections, Table S5: Comparison of other studies using prediction models on different diseases.

## Abbreviations

AML	Automated machine learning
AVG	Average
ICD-10	International Classification of Diseases, Tenth Revision
LightGBM	Light Gradient Boosting Machine
NUTS	Nomenclature of Territorial Units for Statistics
SARIMA	Seasonal Autoregressive Integrated Moving Average
WHO	World Health Organization
XGBoost	Extreme Gradient Boosting
