# Supplementary material for: Time Series Analysis and Forecasting with Automated Machine Learning on a National ICD-10 Database
Source: Int J Environ Res Public Health. 2020 Jul 10;17(14):4979. doi: 10.3390/ijerph17144979 (PMC7400312; doi:10.3390/ijerph17144979)
Supplement: Supplementary file 1 [file ijerph-17-04979-s001.zip › ijerph-836444 - Table S4.docx]

| ****Feature Name**** | ****Var Type**** | ****Unique**** | ****Missing**** | ****Mean**** | ****Std Dev**** | ****Median**** | ****Min**** | ****Max**** | ****Target Leakage**** |
| --- | --- | --- | --- | --- | --- | --- | --- | --- | --- |
| NewCases | Numeric | 829 | 0 | 1716.608 | 630.22 | 1592.5 | 712.0 | 4145.0 | N/A |
| Dates | Date | 132 | 0 | 2013-06-16T08:43:38.181818 | 1159763.17 days | 2013-06-16T00:00:00 | 2008-01-01T00:00:00 | 2018-12-01T00:00:00 | N/A |
| Weekdays | Numeric | 4 | 0 | 21.74 | 0.94 | 22 | 20 | 23 | N/A |
| Days in month | Numeric | 4 | 0 | 30.44 | 0.81 | 31 | 28 | 31 | N/A |
| NUTS2_Hospital_Region | Categorical | 8 | 0 | N/A | N/A | N/A | N/A | N/A | N/A |
