# Supplementary material for: Time Series Analysis and Forecasting with Automated Machine Learning on a National ICD-10 Database
Source: Int J Environ Res Public Health. 2020 Jul 10;17(14):4979. doi: 10.3390/ijerph17144979 (PMC7400312; doi:10.3390/ijerph17144979)
Supplement: Supplementary file 1 [file ijerph-17-04979-s001.zip › ijerph-836444 - Table S5.docx]

| **Author** | **Journal** | **Year** | **Diseases** | **Training Data Period** | **Used Database** | **Database Size** | **Models Trained** | **Forecast Distance** | **National/**  **Regional Scale** | **Metric** |
| --- | --- | --- | --- | --- | --- | --- | --- | --- | --- | --- |
| Brooks LC et al. | PLOS Computational Biology | 2018 | Influenza | 2015/2016  (flu seasons) | CDC (Centers for Disease Control and Prevention), United States | not specified | Combination of different models (not otherwise specified) | Weeks, season | National | Unibin log score, Multibin log score, Absolute error |
| He F et al. | Sci Rep. | 2017 | Influenza | 8,5 years (2006–2015) | Japanese infectious disease  surveillance system | not specified | 2x:  Autoregressive Distributed Lag (ARDL), hybrid model integrating ARDL with  a Generalized Regression Neural Network (GRNN) | 53 weeks | National | RMSE, MAE |
| Lampos V et al. | Sci Rep. | 2015 | Influenza | 5 years  (flue seasons 2008–2013) | CDC (Centers for Disease Control and Prevention), United States | not specified | 7x:  Google flue trends, Elastic net, Gaussian processes in combination with an ARMAX model | Weeks, season | National | Pearson correlation, MAE, MAPE |
| Volkova S et al. | PLOS ONE | 2017 | Influenza-like illness | 3 years  (flue seasons 2011–2014) | Defense Medical Information System (DMIS) | not specified | 3x:  Support Vector Machine (SVM), Ada Boost | Weeks, season | 31 regions | Pearson correlation, RMSE, RMSPE MAPE |
| Huang DC et al. | Sci Total Environ | 2018 | Hand, foot and mouth disease | Approx. 3 years (2009–2011) | Chinese Center for Disease Control and Prevention | not specified | 4x:  Combination of different models (not otherwise specified) | 7 weeks | 1 region | RMSE, Intraclass coefficient (ICC), Pearson correlation |
| Teng Y et al. | PLOS ONE | 2017 | Zika virus | 6 months  (02/2016–08/2016) | Google Trends,  Pan American Health Organization,  WHO | not specified | 2x:  Linear regression model, Autoregressive integrated moving average (ARIMA) | 8 weeks | not specified | Akaike Information Criterion (AIC) |
| Dugas AF et al. | PLOS ONE | 2013 | Influenza | 2004–2011 flu seasons | Urban tertiary  care emergency department (not otherwise specified) | 60,000 adult and 24,000  pediatric patients | 2x:  Generalized linear model (GLM), Generalized Autoregressive Moving Average (GARMA) | 2x seasons | 1 region | Global deviance |
| Chae S et al. | Int. J. Environ. Res. Public Health | 2018 | Chicken Pox, Scarlet Fever, Malaria | 576 days  (01/2016–07/2017) | Korea Center for Disease Control (KCDC) | not specified | 4x:  Deep neural network (DNN),  the long-short term memory (LSTM), autoregressive integrated moving average (ARIMA), the ordinary least squares (OLS) | 7 days | National | RMSE |
| Tian CW et al. | Epidemiology and Infection | 2018 | Hand, foot and mouth disease | 11 years (2008–2018) | National Health  and Family Planning Commission of the People’s Republic of China | not specified | 1x:  Seasonal Autoregressive Integrated Moving Average (SARIMA) | 10 years | National | Determination coefficient, MER (%) |
| Wang H et al. | Epidemiology and Infection | 2017 | Tuberculosis | 13 years (2005–2017) | National Health  and Family Planning Commission of the People’s Republic of China | not specified | 2x:  Seasonal Autoregressive Integrated Moving Average (SARIMA),  SARIMA–GRNN | 12 years | National | Determination coefficient, MER (%) |
