# Supplementary material for: Time Series Analysis and Forecasting with Automated Machine Learning on a National ICD-10 Database
Source: Int J Environ Res Public Health. 2020 Jul 10;17(14):4979. doi: 10.3390/ijerph17144979 (PMC7400312; doi:10.3390/ijerph17144979)
Supplement: Supplementary file 1 [file ijerph-17-04979-s001.zip › ijerph-836444 - Table S3.docx]

| ****Model Name**** | ****Backtest 1 Score (Gamma)**** | ****All Backtests Score (Gamma)**** | ****Holdout Score (Gamma)**** | ****Feature List and Sample Size**** |
| --- | --- | --- | --- | --- |
| AVG Blender | 0.0170 | 0.0246 | 0.0108 | Multiple Feature Lists  4 years 3 months |
| eXtreme Gradient Boosted Trees Regressor with Early Stopping (Gamma Loss) | 0.0184 | 0.0275 | 0.0121 | With Differencing (Average Baseline)  4 years 3 months |
| eXtreme Gradient Boosted Trees Regressor with Early Stopping (Gamma Loss) | 0.0183 | 0.0277 | 0.0125 | With Differencing (Latest)  4 years 3 months |
| Performance Clustered eXtreme Gradient Boosted Trees Regressor | 0.0244 | 0.0287 | 0.0171 | With Differencing (Latest)  4 years 3 months |
| Performance Clustered eXtreme Gradient Boosted Trees Regressor | 0.0217 | 0.0293 | 0.0141 | With Differencing (Average Baseline)  4 years 3 months |
| eXtreme Gradient Boosting on ElasticNet Predictions (Gamma Loss) | 0.0271 | 0.0301 | 0.0153 | No Differencing  4 years 3 months |

Note. AVG, average
