# Supplementary material for: Time Series Analysis and Forecasting with Automated Machine Learning on a National ICD-10 Database
Source: Int J Environ Res Public Health. 2020 Jul 10;17(14):4979. doi: 10.3390/ijerph17144979 (PMC7400312; doi:10.3390/ijerph17144979)
Supplement: Supplementary file 1 [file ijerph-17-04979-s001.zip › ijerph-836444 - suppl. figures final.pdf]

Flowchart of selection of the study sample

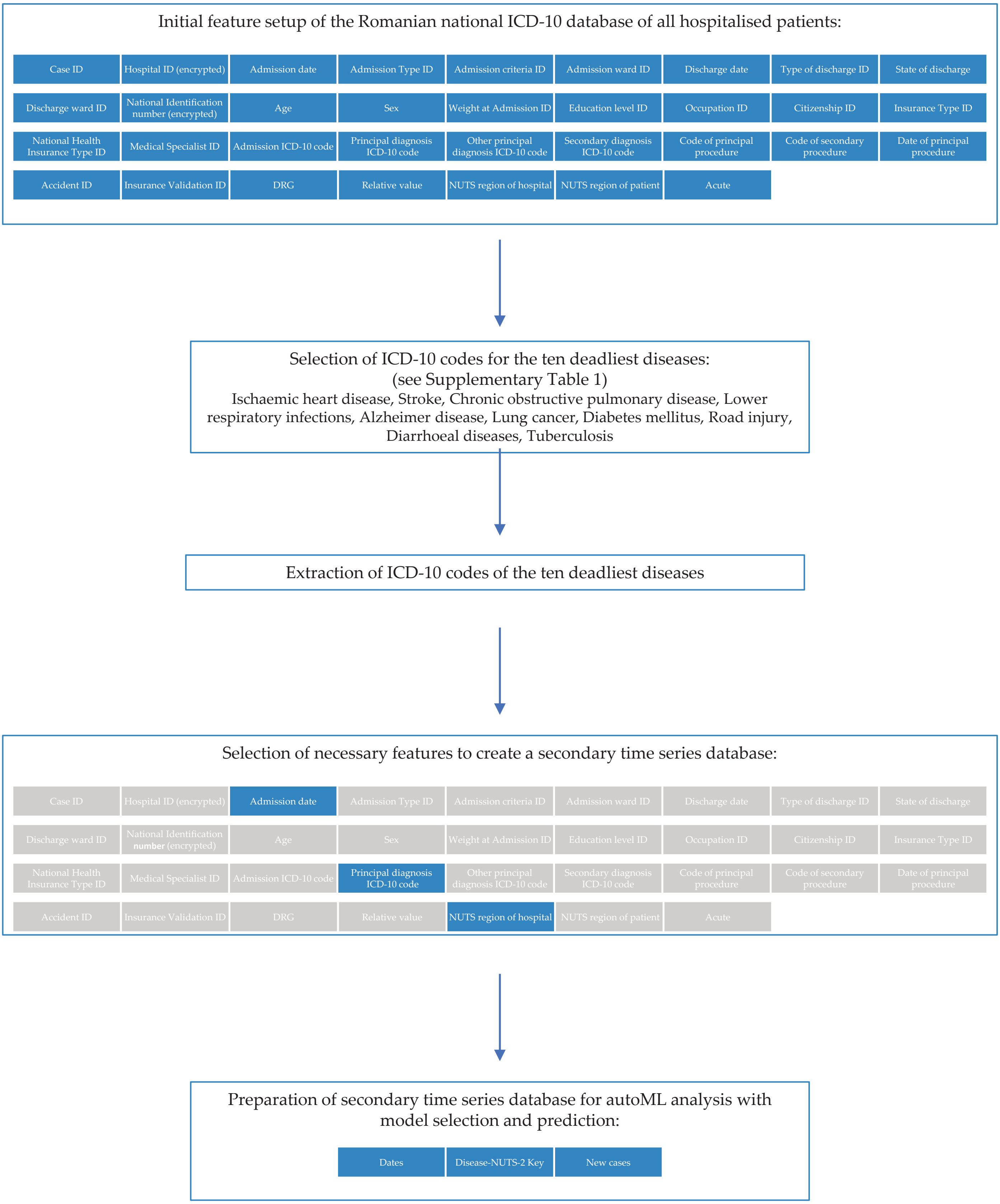

Figure S1.

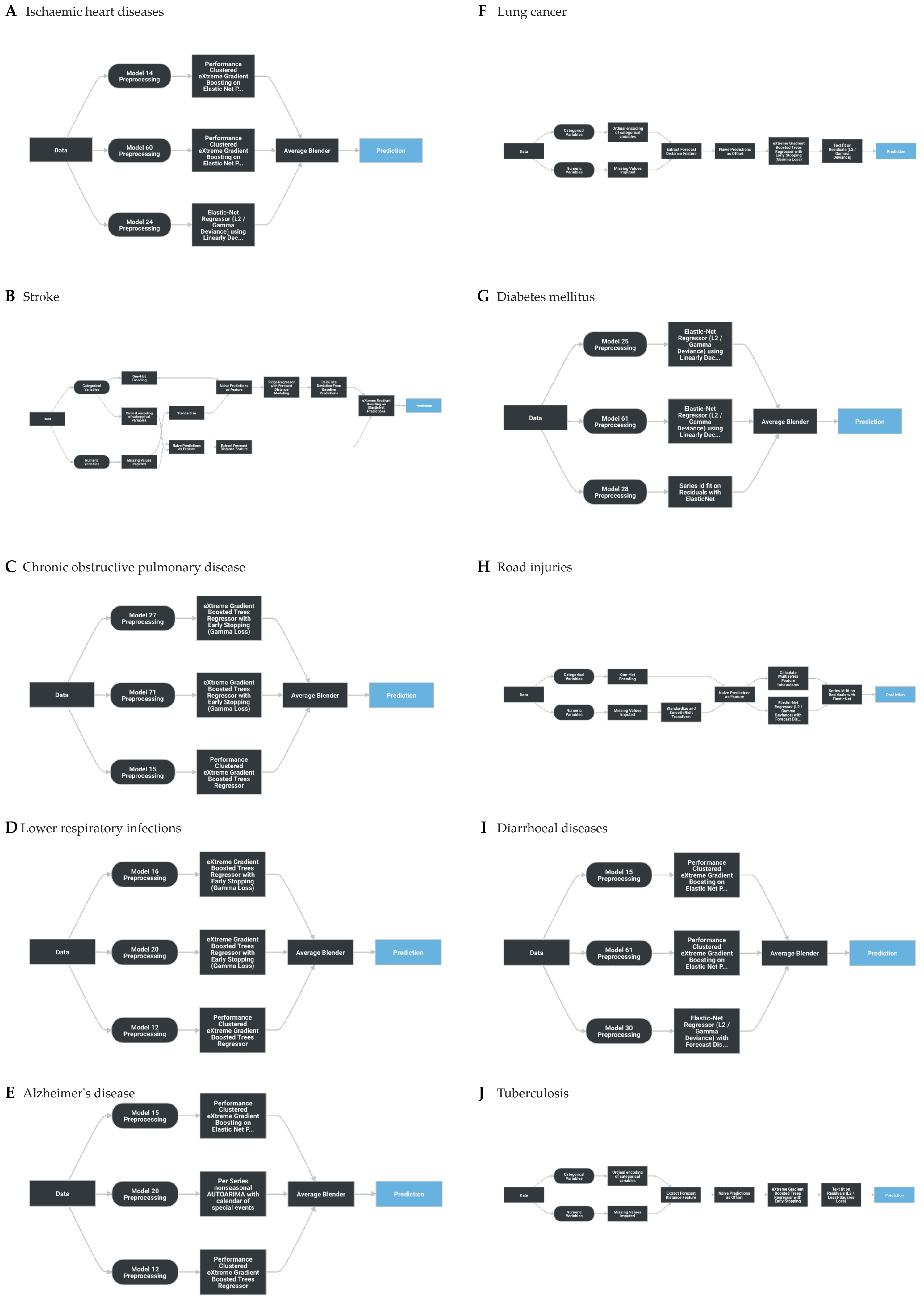
A Ischaemic heart diseases
Data
Model 14 Preprocessing
Model 60 Preprocessing
Model 24 Preprocessing
Performance Clustered eXtreme Gradient Boosting on Elastic Net P...
Performance Clustered eXtreme Gradient Boosting on Elastic Net P...
Elastic-Net Regressor (L2 / Gamma Deviance) using Linearly Dec...
Average Blender
Prediction
F Lung cancer
Data
Categorical Variables
Ordinal encoding of categorical variables
Numeric Variables
Missing Values Imputed
Extract Forecast Distance Feature
Naive Predictions as Offset
eXtreme Gradient Boosted Trees Regressor with Early Stopping (Gamma Loss)
Text fit on Residuals (L2 / Gamma Deviance)
Prediction
B Stroke
Data
Categorical Variables
One-Hot Encoding
Ordinal encoding of categorical variables
Standardize
Naive Predictions as Feature
Ridge Regressor with Forecast Distance Modeling
Calculate Deviation From Baseline Predictions
eXtreme Gradient Boosting on ElasticNet Predictions
Prediction
Numeric Variables
Missing Values Imputed
Naive Predictions as Feature
Extract Forecast Distance Feature
G Diabetes mellitus
Data
Model 25 Preprocessing
Model 61 Preprocessing
Model 28 Preprocessing
Elastic-Net Regressor (L2 / Gamma Deviance) using Linearly Dec...
Elastic-Net Regressor (L2 / Gamma Deviance) using Linearly Dec...
Series Id fit on Residuals with ElasticNet
Average Blender
Prediction
C Chronic obstructive pulmonary disease
Data
Model 27 Preprocessing
Model 71 Preprocessing
Model 15 Preprocessing
eXtreme Gradient Boosted Trees Regressor with Early Stopping (Gamma Loss)
eXtreme Gradient Boosted Trees Regressor with Early Stopping (Gamma Loss)
Performance Clustered eXtreme Gradient Boosted Trees Regressor
Average Blender
Prediction
H Road injuries
Data
Categorical Variables
One-Hot Encoding
Numeric Variables
Missing Values Imputed
Standardize and Smooth Ridit Transform
Naive Predictions as Feature
Calculate Multiseries Feature Interactions
Elastic-Net Regressor (L2 / Gamma Deviance) with Forecast Dis...
Series Id fit on Residuals with ElasticNet
Prediction
D Lower respiratory infections
Data
Model 16 Preprocessing
Model 20 Preprocessing
Model 12 Preprocessing
eXtreme Gradient Boosted Trees Regressor with Early Stopping (Gamma Loss)
eXtreme Gradient Boosted Trees Regressor with Early Stopping (Gamma Loss)
Performance Clustered eXtreme Gradient Boosted Trees Regressor
Average Blender
Prediction
I Diarrhoeal diseases
Data
Model 15 Preprocessing
Model 61 Preprocessing
Model 30 Preprocessing
Performance Clustered eXtreme Gradient Boosting on Elastic Net P...
Performance Clustered eXtreme Gradient Boosting on Elastic Net P...
Elastic-Net Regressor (L2 / Gamma Deviance) with Forecast Dis...
Average Blender
Prediction
E Alzheimer's disease
Data
Model 15 Preprocessing
Model 20 Preprocessing
Model 12 Preprocessing
Performance Clustered eXtreme Gradient Boosting on Elastic Net P...
Per Series nonseasonal AUTOARIMA with calendar of special events
Performance Clustered eXtreme Gradient Boosted Trees Regressor
Average Blender
Prediction
J Tuberculosis
Data
Categorical Variables
Ordinal encoding of categorical variables
Numeric Variables
Missing Values Imputed
Extract Forecast Distance Feature
Naive Predictions as Offset
eXtreme Gradient Boosted Trees Regressor with Early Stopping
Text fit on Residuals (L2 / Least-Squares Loss)
Prediction

Figure S2.

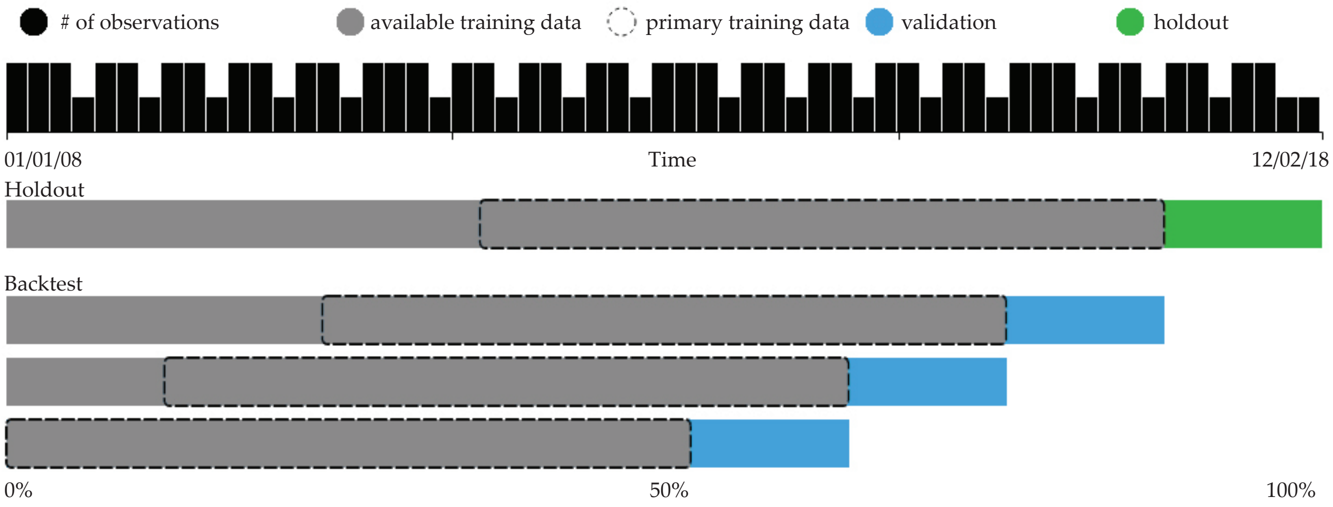
# of observations
available training data
primary training data
validation
holdout
01/01/08
Time
12/02/18
Holdout
Backtest
0%
50%
100%

Figure S3.

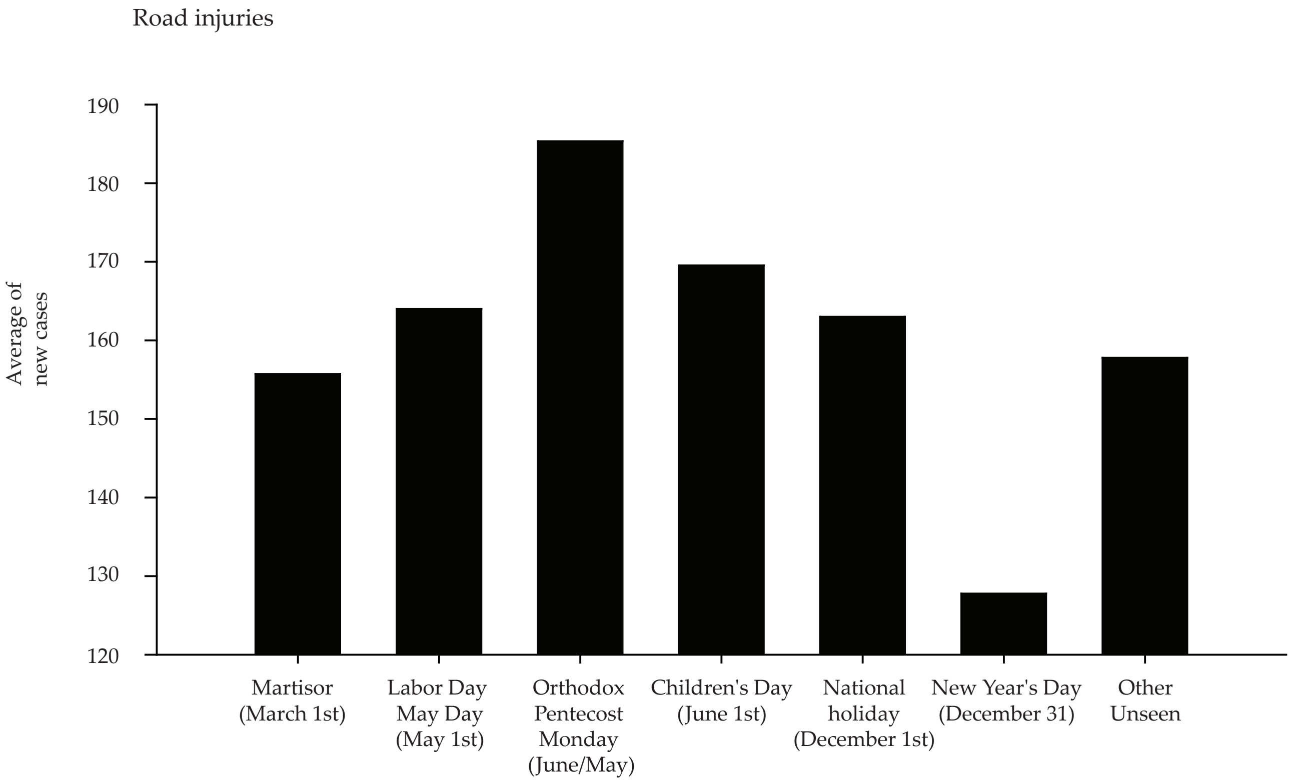
Road injuries
Average of new cases
190
180
170
160
150
140
130
120
Martisor (March 1st)
Labor Day May Day (May 1st)
Orthodox Pentecost Monday (June/May)
Children's Day (June 1st)
National holiday (December 1st)
New Year's Day (December 31)
Other Unseen

Figure S4.
